# Generation of induced pluripotent stem cells from human Tenon’s capsule fibroblasts

**Published:** 2012-11-30

**Authors:** Fei Deng, Huiling Hu, Mengfei Chen, Xuerong Sun, Xiaohong Liu, Zhizhang Dong, Ying Liu, Lei Xi, Jing Zhuang, Jian Ge

**Affiliations:** 1 State Key Laboratory of Ophthalmology, Zhongshan Ophthalmic Center, Sun Yat-sen University, Guangzhou, China; 2 Shengzhen Ophthalmic Center of Jinan University, Shenzhen Eye Hospital, Shenzhen, China

## Abstract

**Purpose:**

This study aimed to develop a feasible and efficient method for generating embryonic stem cell (ESC)-like induced pluripotent stem (iPS) cells from human Tenon’s capsule fibroblasts (HTFs) through the expression of a defined set of transcription factors, which will have significant application value for ophthalmic personalized regenerative medicine.

**Methods:**

HTFs were harvested from fresh samples, and reprogramming was induced by the exogenous expression of the four classic transcription factors, OCT-3/4, SOX-2, KLF-4, and C-MYC. The HTF-derived iPS (TiPS) cells were analyzed with phase contrast microscopy, real-time PCR, immunofluorescence, FACS analysis, alkaline phosphatase activity analysis, and a teratoma formation assay. Human ESC colonies were used as the positive control.

**Results:**

The resulting HTF-derived iPS cell colonies were indistinguishable from human ESC colonies regarding morphology, gene expression levels, pluripotent gene expression, alkaline phosphatase activity, and the ability to generate all three embryonic germ layers.

**Conclusions:**

This study presents a simple, efficient, practical procedure for generating patient-tailored iPS cells from HTFs. These cells will serve as a valuable and preferred candidate donor cell population for ophthalmological regenerative medicine.

## Introduction

Retinal neuron degeneration, such as glaucoma, age-related macular degeneration, diabetic retinopathy, and retinitis pigmentosa, usually results in irreversible retinal cell loss and ultimately leads to an untreatable loss of vision [1]. However, with the rapid advancements in the regenerative medicine field, stem cell-derived replacement therapy has become an attractive alternative option [2]. The development of reprogramming techniques to generate personalized embryonic stem cell (ESC)-like induced pluripotent stem (iPS) cells from somatic cells via the ectopic expression of selected transcription factors [3–5], a process that avoids immunological rejection and ethical difficulties, has opened up new avenues of research in the life sciences [2,6].

iPS cells are artificially reprogrammed into an embryonic-like pluripotent state, capable of unlimited self-renewal and reproduction of all cell types except extraembryonic tissues during the course of their differentiation [3,5,6]. Experimentally, iPS cells are morphologically and functionally indistinguishable from ES cells. Recent reports have shown that iPS cells can be differentiated into neurons [7–9], cardiomyocytes [10], hematopoietic progenitors [11], hepatocyte-like cells [12–14], islet-like cells [15], primordial germ cells [16], and retinal pigment epithelial (RPE) cells [17–19], among others.

To date, human iPSCs have been generated from multiple cellular sources, including skin fibroblasts and keratinocytes, neurons, hepatocytes, gastrointestinal epithelial cells, and mature B/T lymphocytes [20], with variable levels of reprogramming efficiency and technical difficulty. However, no consensus has been reached on the best tissue for harvesting donor cells. Proceeding from the principles of accessibility, susceptibility to reprogramming, and universal availability, many recent studies have been optimistically conducted with skin fibroblasts, lipocytes, and blood cells [21]. However, use of these tissues requires a biopsy or is accompanied by low reprogramming efficiency. Moreover, recent studies have shown that iPSCs may retain cell-of-origin epigenetic memories [22–24] and accumulate other abnormalities as well [25,26].

In light of these issues, the preferred donor cell source should vary according to the specific medical purpose of each iPSC therapy. In ophthalmology, among all of the cell types from which iPSCs can be derived, we consider human fibroblast cells from Tenon’s capsule (HTFs) to be the ideal candidate.

Tenon’s capsule, also called the fascial sheath of the eyeball, is a thin, dense, fibrous membrane between the eyeball and orbital adipose body that ensheathes most of the eyeball, fused with the sclera at the front of the eye and the hard sheath of the optic nerve at the back, forming the cavity within which the eye can move. Because Tenon’s capsule is covered by conjunctiva, the membrane is not readily damaged by external stimulation or prone to accumulating other abnormalities [27,28]. This structure can be easily accessed through a simple surface anesthesia operation involving an incision in the conjunctiva. To our knowledge, excising a small amount of tissue, which would be more than enough to generate personalized iPS cell lines, would not affect the ocular structure or function. Given the accessibility, universality, and mutation resistance of this tissue, evidence that eye-derived HTFs can be easily and efficiently reprogrammed to generate iPS cells would make these cells the preferred cell donor source for ophthalmological therapies. Here, we present a simple method for generating human iPSCs from HTFs. The resulting iPSCs are of excellent quality according to standard criteria.

## Methods

### Collection, culture, and identification of human Tenon’s capsule fibroblasts

**Figure 1:**
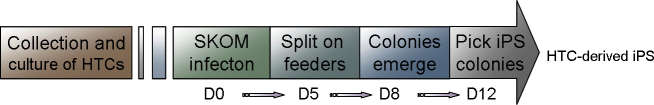
Schematic representation of induced pluripotent stem cell (iPSC) generation from human Tenon’s capsule fibroblasts (HTFs). SKOM refers to the four exogenous factors, Sox2, Klf4, Oct3/4, and c-Myc.

HTFs were obtained anonymously from tissue explants taken during strabismus or glaucoma-filtering surgery or from the eye bank (donors aged 18 to 60 years) at the ZhongShan Ophthalmic Center, Sun Yat-sen University, with the approval of the ethical committee of the ZhongShan Ophthalmic Center and in accordance with the Declaration of Helsinki. We thoroughly reviewed the donors’ clinical records and excluded patients with ocular surface diseases and systemic conditions such as infections or diabetes. Operations were performed using a standardized surgical technique under sterile conditions according to the protocols described previously with minor modifications. Briefly, conjunctiva and Tenon's capsule were carefully separated by subconjunctival anesthesia. After a conjunctival flap incision was made about 1 mm from the limbus, Tenon’s capsule was opened by blunt dissection of the conjunctiva and Tenon tissue. Small samples of approximately 3×3-mm Tenon's capsule tissues were excised near the fornix with relative hypertrophy of organization and no apparent vessels. Thereafter, the conventional steps of the strabismus or glaucoma-filtering surgery procedure were followed. The tissues were immediately transferred into tubes of normal saline solution and cut into approximately 2-mm pieces under sterile conditions. The tissue samples were seeded in 60-mm culture dishes and maintained in Dulbecco’s modified Eagle’s medium (DMEM, Gibco, Grand Island, NY) containing 10% fetal bovine serum (FBS, Invitrogen, Auckland, NZ), and 1% penicillin/streptomycin (Invitrogen) was supplemented during the early stages of culture. About 8 days after seeding, primary cultures were passaged at 50% confluence with 0.25% trypsin-EDTA (Invitrogen) into 100-mm culture dishes. For subsequent passaging, cells were dissociated using 0.25% trypsin-EDTA (Invitrogen) at 90% confluence, and replated at 1:2 or 1:3 dilutions at a concentration of 1×10^6^ cells per 100-mm plate. HTFs were characterized by their adherent morphology (phase contrast microscopy) as well as the expression of vimentin and fibronectin (immunofluorescence; Figure 1B). Cells from passages 3 to 5 were used for the following experiments.

### Generation and culture of human Tenon’s capsule fibroblast-derived induced pluripotent stem cells

iPS cells were induced from HTFs by the exogenous expression of four transcription factors according to the scheme in Figure 1A. Retroviruses were generated by transfecting 293FT cells (Invitrogen, Cat. R700–07) using FuGENE HD (Roche, Indianapolis, IN) with reprogramming factors (pMXs-based retroviral vectors; Addgene) containing cDNAs for Sox2, Klf4, Oct3/4, and c-Myc (SKOM). Briefly, 293FT cells (Invitrogen) were seeded at 1.5×10^6^ cells per 60-mm dish and incubated overnight. The next day, the cells were transfected with pMXs vectors along with the retrovirus packaging vectors VSV-G and GP (pVPack Vectors, Stratagene, La Jolla, CA) with FuGene HD (Roche). The transfection cocktail per individual vector used was 300 μl opti-MEM (Invitrogen), 4 μg reprogramming factor, 2.5 μg GP, 1.5 μg VSV-G, and 20 μl Fugene HD (Roche), according to the manufacturer’s instructions. The medium was replaced with 4 ml fresh medium after overnight incubation. About 48 and 72 h after transfection, the viral supernatants were harvested and then mixed for filtering through a 0.45 μm low protein binding cellulose acetate filter (Millipore, Billerica, MA). Subsequently, the retrovirus mixture was concentrated for 10 folds using Amicon Ultra-15 Centrifugal Filter Units (Millipore). HTFs were seeded on six-well dishes (approximately 75,000 cells per well) one day before transduction, infected with freshly concentrated viral supernatants supplemented with 4 mg/ml Polybrene (Sigma-Aldrich, St. Louis, MO) to increase the infection efficiency. A green fluorescent protein retrovirus was used as a control to monitor infection efficiency. Twenty-four hours after infection, the medium was replaced with fresh 20% FBS-DF12/DMEM (DF12 with 20% FBS, 10 ng/ml basic fibroblast growth factor, 1% nonessential amino acids, 1% L-glutamine, 0.1 mM β-mercaptoethanol, 100 U/ml penicillin, and 100 mg/ml streptomycin [all from Gibco, Invitrogen]). On day 5 or 6, cells were trypsinized at 90% confluence, transferred onto mitomycin C-inactivated mouse embryonic fibroblast (MEF) feeder cells in a 100-mm culture dish (approximately 40,000 cells per dish), and supplied with fresh human ESC medium each day (substituting FBS with Knockout Serum Replacement; Invitrogen). Between 12 and 16 days after infection, iPSC colonies were mechanically picked and expanded in human ESC medium on feeders and further passaged by mechanical dissociation to small clones at a ratio of 1:2 or 1:3 approximately once a week. For subsequent cultures, Plasmocin (InvivoGen, San Diego, CA) was added to inhibit the growth of mycoplasma. All of the cells used in this study were cultured in a 37 °C humidified incubator containing 5% CO_2_. Three random representative HTF-derived iPSC lines (TiPS), TiPS clone 3 (TiPS-C3), TiPS clone 5 (TiPS-C5), and TiPS clone 13 (TiPS-C13), were used for further analyses.

### Real-time polymerase chain reaction

Total RNA was isolated using the TRIzol Reagent (Ambion, Austin, TX) and subjected to the SuperScript III One-Step RT–PCR System with Platinum Taq High Fidelity (Invitrogen), according to the manufacturer’s instructions. Real-time PCR analysis was performed using an ABI 9700 GeneAmp System and SYBR Green reagents (Applied Biosystems), normalized by TATA box binding protein (TBP) levels, and compared to positive/negative control samples. Primer sequences are listed in Table 1.

**Table 1:** Primer sequences used in real-time PCR.

Gene	Forward primer	Reverse primer
*T-OCT4*	GAGGAGTCCCAGGACATCAA	TGGCTGAATACCTTCCCAAA
*E-OCT4*	TGTACTCCTCGGTCCCTTTC	TCCAGGTTTTCTTTCCCTAGC
*T-NANOG*	TACCTCAGCCTCCAGCAGAT	CCTTCTGCGTCACACCATT
*E-NANOG*	CAGTCTGGACACTGGCTGAA	CTCGCTGATTAGGCTCCAAC
*T-SOX2*	AGCTACAGCATGATGCAGGA	GGTCATGGAGTTGTACTGCA
*E-SOX2*	GCTAGTCTCCAAGCGACGAA	GCAAGAAGCCTCTCCTTGAA
*T-CMYC*	ACTCTGAGGAGGAACAAGAA	TGGAGACGTGGCACCTCTT
*E-CMYC*	CGGAACTCTTGTGCGTAAGG	CTCAGCCAAGGTTGTGAGGT
*T-KLF4*	CCCAATTACCCATCCTTCCT	ACGATCGTCTTCCCCTCTTT
*E-KLF4*	TATGACCCACACTGCCAGAA	TGGGAACTTGACCATGATTG
*TBP*	AACCACGGCACTGATTTT	CTGCCAGTCTGGACTGTTCT

### Immunofluorescence

TiPS and hES cells grown on feeder cells were fixed in 4% paraformaldehyde/PBS (NaCl 8, KCl 0.2, Na_2_HPO_4_•12H_2_O 2.8, KH_2_PO_4_ 0.2 g/l; pH 7.4; Sigma-Aldrich) for 15 to 20 min, permeabilized with 0.1% Triton X-100 in PBS (Sigma-Aldrich) for 10 min, blocked in 4% normal goat serum (Boster Biologic Technology, Wuhan, China) for 30 min, and incubated with primary antibodies in 4% normal goat serum overnight at 4 °C in the dark. The next day, the cells were washed three times with PBS and incubated with secondary antibodies (1:200) and DAPI (Boster Biologic Technology) in PBS for 30 min to 1 h at room temperature in the dark. Antibody SSEA-4-PE (Millipore) was used at 1:100 dilution. Primary antibodies TRA-1–60 (Millipore), OCT-4 (Millipore), and NANOG (Cell Signaling, Danvers, MA) were used at 1:200 dilution, and Cy3- or Alexa 488–conjugated goat anti-mouse immunoglobulin G (Invitrogen) was used as the secondary antibody. Fluorescent confocal images were acquired with a laser-scanning microscope (LSM 510; Carl Zeiss, Thornwood, NY).

### Flow cytometry analysis

Before flow cytometry detection, TiPS and hES were passaged by mechanical dissociation and maintained on six-well plates coated with Matrigel (Becton Dickinson, Franklin Lakes, NJ) in mTeSR1 (Stem Cell Technologies, Vancouver, BC, Canada) for feeder-free culture. HTF was used for the negative control group. Three days after the subculture, TiPS, hES, and HTF were separately trypsinized into single cell suspensions. Cells (1×10^6^ cells) were fixed in 4% paraformaldehyde/PBS for 15 min, stained with anti-SSEA4-PE (Millipore, 1:100) for 1 h at 4 °C in the dark, washed twice with PBS, resuspended in PBS buffer, and then analyzed with flow cytometry (FACS Aria; BD Biosciences).

### Alkaline phosphatase activity

Alkaline phosphatase activity was detected with the Quantitative Alkaline Phosphatase ES Characterization Kit (Millipore, cat. SCR066) according to the manufacturer’s recommendations.

### Teratoma assay

To test pluripotency, human iPS cells (approximately 1×10^6^ TiPS cells in 10 μl DMEM/F12 medium) were subcutaneously injected into the dorsal flanks of NOD-SCID mice. The hESC was used as a positive control. NOD-SCID mice were obtained from the Center of Experimental Animals at Sun Yat-sen University, China. Ethical approval was obtained before the start of the study from the Council on Animal Care and Use Subcommittee at the Zhongshan Ophthalmic Center, Sun Yat-sen University (ID: SYXK2010–0058). About 6 weeks after injection, tumors formed from TiPS clones and hESC clones. We dissected tumors by 8 weeks and analyzed the tissues with hematoxylin and eosin staining.

### Statistical analysis

All analyses were performed in triplicate, and the data are presented as the mean ± standard deviation (SD). The data were analyzed with the Student *t* test using SPSS 13.0 (Chicago, IL), with p<0.05 considered significant.

## Results

### Extraction and characterization of human Tenon’s capsule fibroblasts

**Figure 2:**
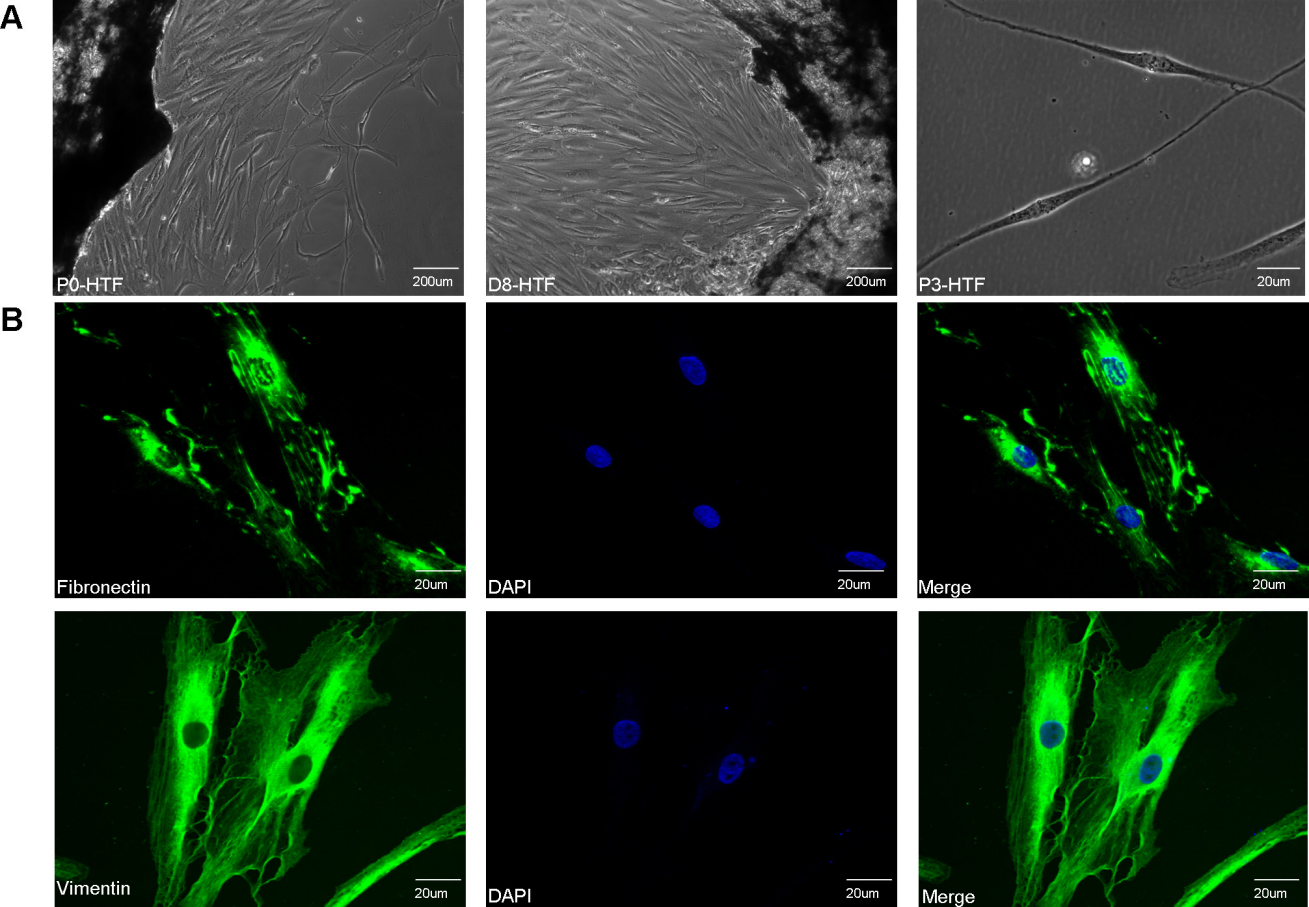
Extraction and characterization of human Tenon’s capsule fibroblasts. **A**: Phase contrast images of human Tenon’s capsule fibroblasts (HTFs). Generally flat, elongated, spindle-shaped cells climb out of the tissue approximately 1 week after seeding. Scale bar=200 μm **B**: Confocal immunofluorescent images of HTFs. Expression of fibronectin and vimentin. Scale bar=20 μm.

Approximately 1 week after seeding, cells grew from the edge of original 2×2-mm explant, and they overspread after 2–3 weeks (Figure 2A). For subsequent passages, cells were dissociated using 0.25% trypsin-EDTA (Invitrogen) at 90% confluence, and replated at 1:2 or 1:3 dilutions at a concentration of 1×10^6^ cells per 100-mm plate. The HTFs in-vitro culture grew in monolayers and exhibited homogeneous morphology of a zonary or spindly, generally flat, elongated shape (Figure 2A). Further, all fibroblast types were shown to be positive for fibronectin and vimentin as assessed with immunocytochemical staining (Figure 2B). Six samples were obtained, and six individual cell strains were developed from these tissues, dubbed HTF-1 to −6, three of which (HTF-1, −3, and −5, randomly chosen) were used for the cell experiments in this study.

### Generation of human Tenon’s capsule fibroblast–derived induced pluripotent stem cells from human Tenon’s capsule fibroblasts

**Figure 3:**
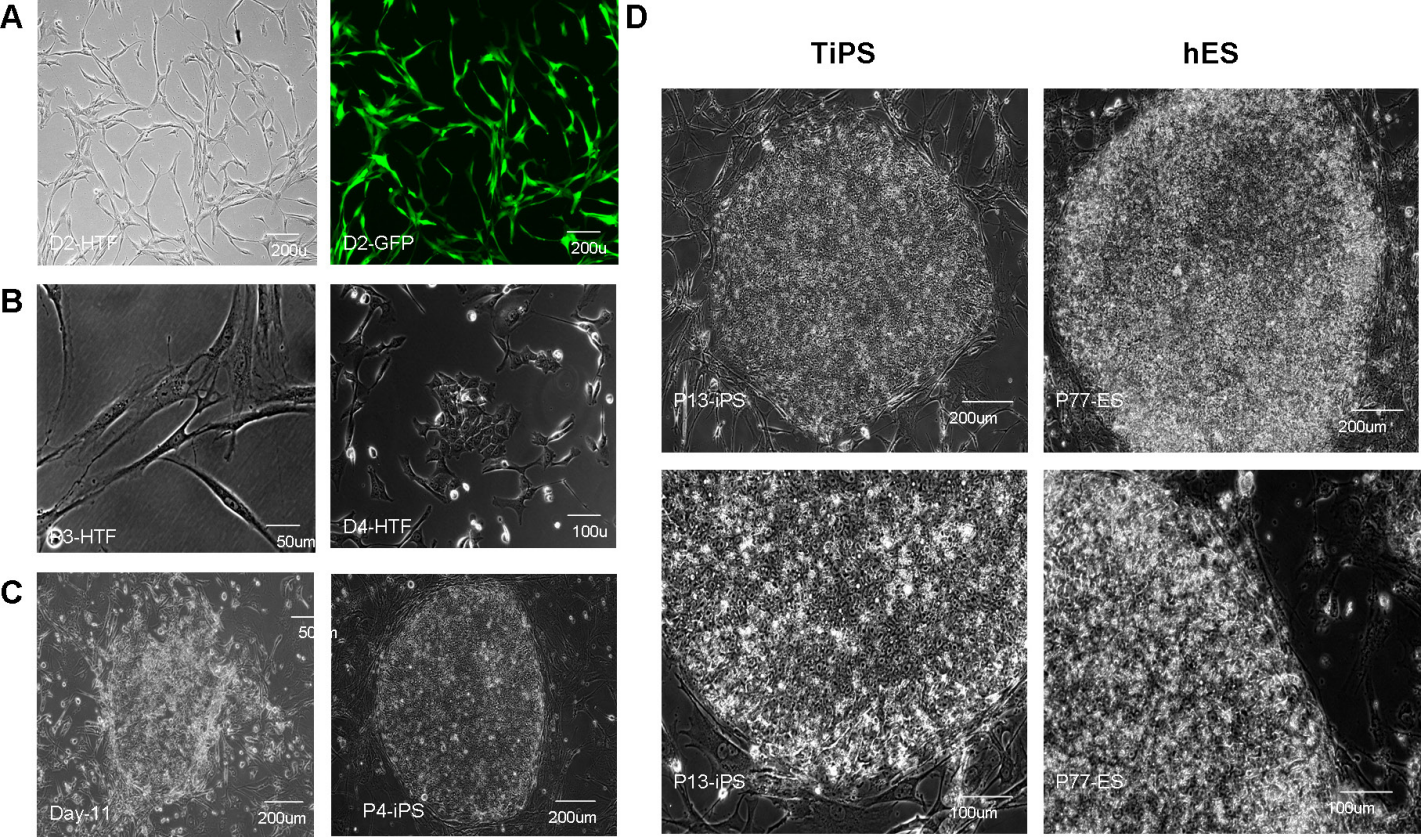
Generation of induced pluripotent stem cells from human Tenon’s capsule fibroblasts. **A**: Immunofluorescent image of human Tenon’s capsule fibroblasts (HTFs) 24 h after infection with a control green fluorescent protein (GFP) vector. An infection efficiency of nearly 100% was observed in cells infected with the control GFP vector. Scale bar=200 μm **B**: Phase contrast image of mesenchymal-epithelial transition (MET). During the early stages of reprogramming, fibroblasts underwent a morphological transformation from a mesenchymal to an epithelial-like morphology. Scale bar=50 μm and 100 μm, respectively. **C**: Phase contrast image of granulated and human embryonic stem cell (hESC)-like colonies. The typical hESC-like colony was flat, with well defined phase-bright borders. Scale bar=200 μm. **D**: Phase contrast images of an established induced pluripotent stem (iPS) cell line at passage P 13 and a control hES cell line at passage P 77. Both include typical honeycomb cells with a high nucleus-to-cytoplasm ratio and prominent nucleoli. Scale bar=200 μm and 100 μm, respectively.

Twenty-four hours after infection, green fluorescent protein–expressing vectors demonstrated nearly 100% infection efficiency with immunofluorescence analysis (Figure 3A). After approximately 3–4 days, cells infected with the reprogramming vectors began to undergo a dramatic morphological mesenchymal-epithelial transition (MET; Figure 3B), a critical reprogramming sign initiated before pluripotent markers are reactivated. Approximately 8–11 days after infection, we observed the emergence of several granulated colonies dissimilar to hESCs in morphology (Figure 3C). Between days 12 and 14, hESC-like colonies appeared. These colonies presented a single flat layer and well defined phase-bright borders; within the colonies, we observed honeycomb-shaped cells with a high nucleus-to-cytoplasm ratio and prominent nucleoli (Figure 3D). Approximately 15–20 identifiable hESC-like colonies from 4×10^4^ transduced HTFs were picked mechanically (which reached about 0.05% of parental cells, consistent with previous reports) and expanded in human ESC medium on feeders.

### Human Tenon’s capsule fibroblast–derived induced pluripotent stem cells express human embryonic stem cell–specific genes and markers

**Figure 4:**
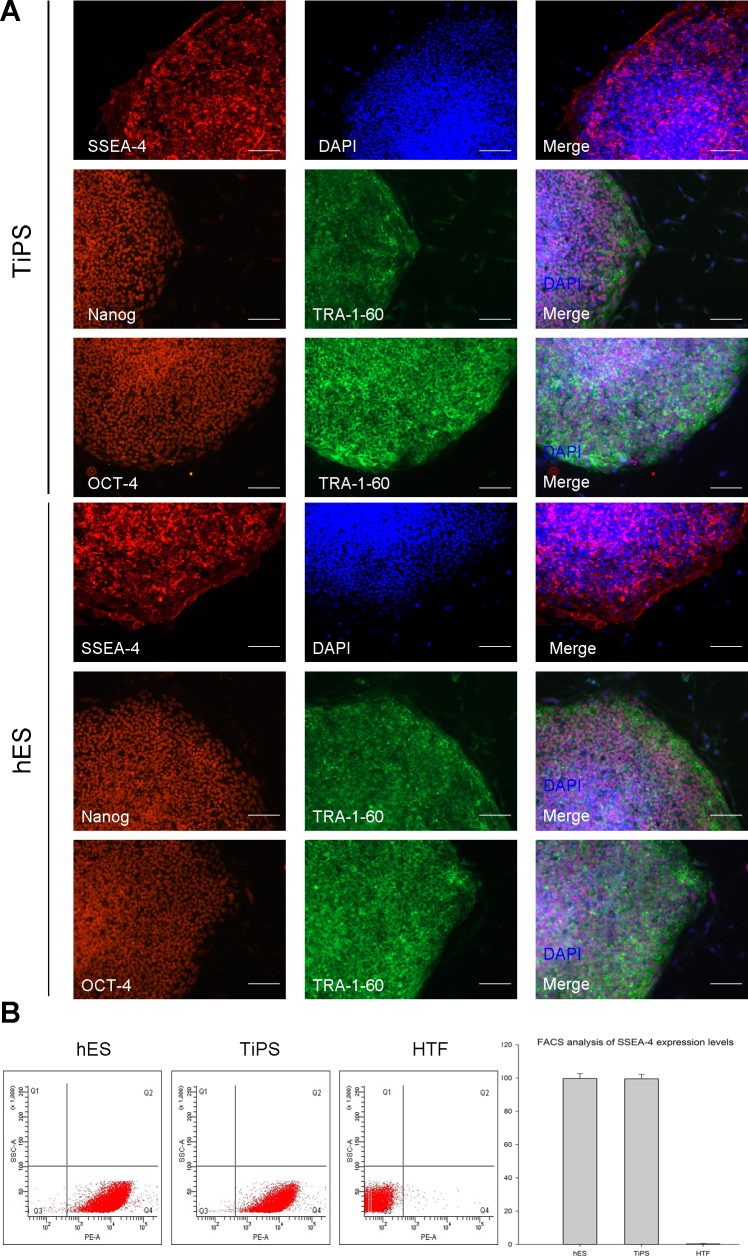
Expression of human embryonic stem cell markers in human Tenon’s capsule fibroblast derived induced pluripotent stem cells. **A**: Confocal immunofluorescent images of representative human Tenon’s capsule fibroblast derived induced pluripotent stem cells (TiPS) clones stained with the human embryonic stem (hES) markers SSEA-4 (red), TRA-1–60 (green), Nanog (red), and Oct-4 (red). Nuclei were stained with DAPI (blue). hESCs were used as a control. Scale bars=50 μm. **B**: Flow cytometry analysis of the equivalent SSEA-4 expression levels in TiPS and hESCs. 99.5% and 99.7% SSEA-4 + cells (in the Q4 area), respectively, whereas 0.3% in HTFs (p<0.001).

**Figure 5:**
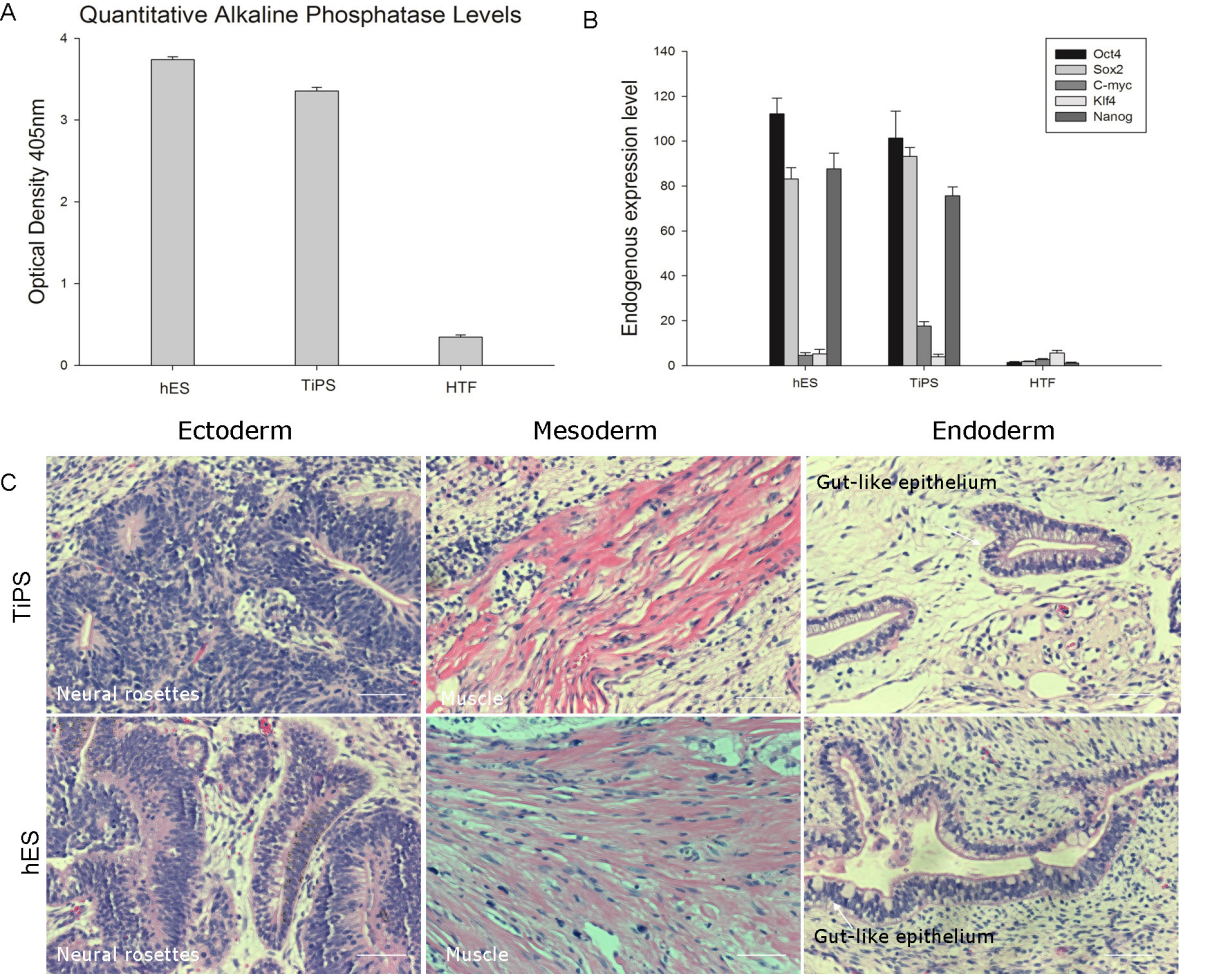
Pluripotency and differentiation potency of human Tenon’s capsule fibroblast derived induced pluripotent stem cells. **A**: Comparable alkaline phosphatase activity levels were observed in human Tenon’s capsule fibroblast derived induced pluripotent stem (TiPS) cells and human embryonic stem cell (hESC). The graph shows the mean±SD (n=3). **B**: Quantitative real-time RT–PCR analysis of endogenous Oct4, Sox2, c-Myc, Klf4, and Nanog expression in TiPS clones, hESC clones, and HTFs. Nanog, Oct3/4, and Sox2 were expressed at a higher level in TiPS clones and hESC clones, whereas Klf4 and c-Myc were expressed at a lower level, indicating silencing. Transcript levels were normalized to TBP levels. The graph shows the mean±SD (n=3). **C**: Teratoma derived from TiPS and hESC. Hematoxylin and eosin staining of the TiPS-derived teratoma, showing derivatives of the three germ layers, including neural rosettes (ectoderm), muscle (mesoderm), and gut-like epithelium (endoderm). hESCs were used as a control. Scale bar=50 μm.

We randomly selected two representative induced clones and compared the expression of key markers and genes with H9 human ES cells. Confocal immunofluorescence analysis revealed that the pluripotency markers SSEA-4, TRA-1–60, OCT-4 and NANOG were highly expressed in both cell types (Figure 4A). Equivalent SSEA-4 expression levels were also demonstrated through FACS analysis, 99.5% and 99.7% SSEA-4 + cells in TiPS and hESCs respectively, whereas 0.3% in HTFs (Figure 4B). Analyses of alkaline phosphatase activity likewise detected similar activity levels between the TiPS clones and the hESCs (Figure 5A). A real-time PCR analysis with primers targeting endogenous genes (OCT-4, NANOG, SOX-2, KLF-4, and C-MYC) revealed that the gene expression patterns of the TiPS clones were largely similar to those of the hESCs: Nanog, Oct3/4, Sox2 were expressed at a higher level in TiPS clones and hESC clones, whereas Klf4 and c-Myc were expressed at a lower level, indicating silencing (Figure 5B). These results provide strong evidence that the HTF-derived iPSC colonies were indistinguishable from human ES cells.

### Human Tenon’s capsule fibroblasts–derived induced pluripotent stem are pluripotent and can differentiate into the three germ layers

We observed tumor formation in NOD-SCID mice injected with TiPS cells or hES cells approximately 6 weeks after injection. We dissected the tumors by 8 weeks and analyzed the tissues with hematoxylin and eosin staining. A histological examination revealed that the tumors contained tissues from all three germ layers (Figure 5C), including gut-like epithelial tissues (endoderm), muscle (mesoderm), and neural tissues (ectoderm).

## Discussion

In our study, we demonstrated that iPS cells can be efficiently generated from adult human Tenon’s capsule fibroblasts. The established human TiPS cells closely resemble hESCs according to standard procedures including ES cell–like morphology, immunofluorescence for human ESC markers, alkaline phosphatase activity, and real-time PCR for endogenous ESC gene expression. Furthermore, these cells maintain the capacity to differentiate into cell types of the three germ layers (ectoderm, mesoderm, and endoderm) in teratomas after subcutaneous injection into nonobese diabetic–severe combined immunodeficient mice.

Somatic cell-derived and patient-tailored iPS cells, potentially the equivalent of ESCs, possess major potential advantages for practical cell transplantation, as these cells sidestep ethical concerns and immune reactions. To date, various somatic cell types have been successfully reprogrammed, though with variable induction efficiencies and associated technical difficulties [20]. However, no consensus has been achieved regarding the best type of donor cells. To proceed with iPS cells for clinical application, these donor cells should be easily accessible and well cultured, and their method of isolation should be as noninvasive as possible [29].

Our study has shown HTFs are an ideal candidate for cell research in vitro due to the convenience of culturing and stable proliferativity. In addition, the fact that HTFs can be acquired from patients of any age, sex, body condition, or ethnic group under strict operation standards and aseptic conditions during ophthalmic surgery makes the derived TiPS more practical for subsequent clinical applications.

We obtained 15–20 iPS cells colonies from 4×10^4^ transduced HTFs. The efficiency of HTF reprogramming is comparable with that of human adipose stem cells and dermal fibroblasts [4,30], but HTF isolation is steady and minimally invasive regarding clinical ophthalmological applications.

In particular, the evidence from our findings that iPS cells endogenously express SOX-2 with high levels, which has been shown as a key regulatory gene that functions throughout retinal development [31–33], indicates that these cells may be particularly valuable for retinal regeneration research. As a relatively immunologically isolated compartment compared to other parts of the body, the eye is an ideal target for cell replacement therapy [34]. Cells can be injected directly, and changes in the retina can be measured non-invasively just by peering into it. In November 2010, stage I hES clinical trials worldwide, to test the safety of implanting hESC-derived retinal cells into the eye to treat certain types of blindness, were approved by the USA Food and Drug Administration (FDA). Meanwhile, to make ophthalmological application of iPS cells a reality, a Japanese group plans to cure blindness in patients with macular degeneration within the next 3 years using iPS-derived RPE transplants. These trials represent the first halting steps toward new avenues of regenerative medicine that may fundamentally transform the prevailing dogma [35].

Some donor cell types may be more prone than others to accumulate epigenetic imprints and somatic cell mutations. In the eye, compared with corneal/lacrimal gland-derived fibroblasts, Tenon’s capsule-derived fibroblasts suffer fewer somatic cell mutations and copy number variations due to less direct exposure to the external environment and inflammatory stimuli [36], making this cell type an attractive candidate for efficiently and safely generating patient-tailored iPS cells. Moreover, the inducible differentiation capacity of different iPSC lines has been shown to vary by cell line and to be influenced by epigenetic memory. It has been reported that iPSC lines from the eye spontaneously differentiate into RPE more readily than iPSC lines derived from foreskin [19]. The exact molecular mechanism underlying the variable differentiation capacity, however, remains somewhat undefined. However, if it is true that iPSC lines retain a memory of their donor tissue, then iPSC lines from the eye should be more suitable for ophthalmological transplantation or eye disease modeling than other iPSC lines.

However, clinical application of iPSC remains threatened by a major technical hurdle of genomic integration by retroviral vectors [37–39]. Viral DNA or oncogene incorporation into chromosomes can probably cause insertional mutagenesis, lead to disruption of gene transcription, and induce malignant transformation, which constitutes a major safety concern in cell replacement therapy. Finally, small molecules or other alternative methods of transgene delivery for avoiding genomic integration will be required. Nevertheless, current retroviral reprogramming methods can be immediately applied in establishing disease models for drug screening and toxicity testing [29].

However, we are still a long way from the final targets to realize the clinical application of TiPS. Further research is needed to optimize the generation, and directed differentiation of TiPS to retinal cells is necessary before intraocular transplantation.

In conclusion, we present a preliminary method for efficiently generating patient-specific TiPS of excellent quality according to standard criteria. We found that HTFs were useful donor cells for producing patient-specific iPS cells because the primary cells are accessible and universally available, can be harvested under acceptable and aseptic conditions, contain fewer mutations, and are susceptible to reprogramming. TiPS represent promising materials for retinal regeneration applications, especially given their source and endogenous expression of SOX-2. Therefore, these cells possess the potential to be used in future cell transplantation therapies for patients with retinal neurodegenerative diseases, which may pave the way for vision rehabilitation.
